# A modified Delphi method to elicit and compare perceptions of industry trends

**DOI:** 10.1016/j.mex.2020.101081

**Published:** 2020-09-28

**Authors:** Kathrine Friis-Holm Egfjord, Kristian J. Sund

**Affiliations:** Roskilde University, Denmark

**Keywords:** Delphi method, Cognitive mapping, Business model innovation, Incumbent firms, Industry trends, Issue interpretation

## Abstract

Existing literature suggests that one reason why incumbent firms fail at radical business model innovation is the existence of cognitive barriers, such as a dominant core business logic. Such a dominant logic may result in organizational tensions, when a new logic emerges. In a related article in *Technological Forecasting & Social Change*, we argue that differences in strategic issue identification and interpretation can help to explain the cognitive barriers in this context. In the present article, we propose and demonstrate a 7-step Delphi based method to elicit and examine differences in the perception of industry trends, comparing innovators, core business employees, and external experts. We use the case study of a leading Nordic insurance firm to illustrate the method.

Therefore, in this article, we:

• Suggest that differences in strategic issue identification and interpretation can explain the cognitive barriers that emerge when incumbent firms try to engage with radical business model innovation.
• Propose a Delphi-based method to elicit and examine differences in the perception of industry trends, comparing innovators, core business employees, and external experts.
• Demonstrate the method on a case firm from the insurance industry, in a way that can easily be replicated in future studies.

## Graphical abstract

**Figure:**
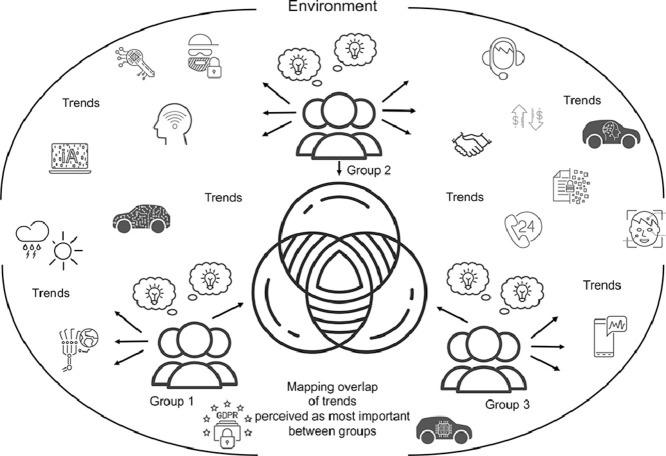

Specifications Table

**Table:** 

Subject Area	Management Science Social Science
More specific subject area	Innovation studies
Method name	*Delphi method; Knowledge elicitation technique*
Name and reference of original method	Schmidt, R. C. (1997). Managing Delphi surveys using nonparametric statistical techniques. Decision Sciences, 28(3), 763–774; Okoli, C., & Pawlowski, S. D. (2004). The Delphi method as a research tool: An example, design considerations and applications. Information & Management, 42(1), 15–29.
Resource availability	Not Applicable

## Method details

Incumbents, defined as mature firms with a strong position in the market, tend to fail at more radical business model innovation [1,2]. Faced with environmental changes, such firms must continuously adjust to shifting market conditions**.** While much research on business models pays attention to start-ups and their creation of new business models, a much smaller part focuses on incumbent firms, with already existing ones. The situation of incumbent firms is unique, as they unlike start-ups, are in a position where they have to balance the exploration of new business models with the exploitation of existing ones [1,^3^,^4^,5]**.** A common way of organizing such exploration is to establish a dedicated innovation team, department or unit within the organization, with the tasks of monitoring environmental trends and generating new ideas**.** However, managers in the core business are likely to resist such exploration if they believe it threatens the existing business. If the new business model does not immediately fit the “dominant logic” of the core business, there is a risk that new ideas are discarded [1,^2^,^6^,^7^,8]**.**

In existing business model innovation research, a number of studies have pointed out cognition as playing a role in enabling or restricting innovation, but the literature has been criticized as lacking explanatory mechanisms [9]. What we do know is that the business model can be studied as a form of cognitive structure, mental map, or schema, of how a firm creates value [10,11], and that managers’ cognitions and sense-making can influence business model design [12]. Individuals in organizations collectively act as interpretation systems, (1) sensing and sharing information about the environment, (2) interpreting this information on behalf of the organization, and (3) devising appropriate strategies and actions in response to these interpretations [13,14]. However, only information that is available and which is perceived as relevant has the potential to be interpreted and acted upon. In the context of business model innovation in incumbent firms, innovators are often placed in their own separate department with tasks that include interpreting changes in the environment. The information that they interpret and perceive as relevant will be converted into recommendations for changes to existing business models or proposals for completely new business models. The information environment in which they operate will naturally be different from that of managers and employees in the core business. Without a shared perception and interpretation of information, the suggestions developed by innovators can be perceived as irrelevant to members of the core business. In other words, if innovators in an incumbent firm possess a different perception of the world than employees from the core business, they will come up with solutions to the “wrong” problems and therefore face resistance.

Numerous methods have been used in the managerial and organizational cognition literature to elicit managerial cognitions (for an overview see e.g. [15]). For example, open interview techniques have been used to elicit cognitive constructs. Causal mapping techniques have been used to link such constructs into mental maps. Policy capturing techniques have been used to create decision-making scenarios. Finally, repertory grid analysis has combined structured interviews with grid-based ratings to elicit a person's personal constructs for given topics. Common to these methods is that they tend to focus on individual rather than group cognition, and that they tend to be time-consuming to conduct. Methods based on mathematical simulation can yield interesting results as well, but involve hypothetical rather than real constructs, making them inappropriate for our study on shared perceptions.

In a recent article in *Technological Forecasting & Social Change* [16] we presented a novel way to measure differences in perceptions between groups in an organization, based on a modified Delphi method. We will here elaborate on this method, which contributes to the literature on cognition elicitation. Our objective was to elicit the perceptions of groups of individuals, rather than simply of one individual. Existing knowledge elicitation methods from studies of cognition have typically focused on individual level cognition, but in the organizational context decision-making usually takes place at the group level, as an outcome of a sense-making process such as described earlier. If this is the case, it is useful to emulate such a process, which is exactly where the Delphi method can offer a useful methodological approach.

The Delphi method is one of the most widely used techniques for technological forecasting. The method was developed by the American research institute Rand Corporation in the 1950s to find the best defense system for the United States against the Soviet Union. Later, it has gained ground in social science and business, as a way to solicit expert opinions (see e.g. [17]). The basic principles of the method are anonymity, repetition, controlled feedback and group response. Typically, the goal is to achieve the most reliable consensus on a given topic among a group of experts. The study is usually carried out in the form of a questionnaire conducted on a panel composed of experts in the field. The method has been used in many different ways and is often combined with other methods [18]**.**

Our purpose with the Delphi method was to emulate a sense-making process between experts who are not physically together, and do so in a time-efficient manner. Consistent with the Delphi method, we wished to develop separate and reliable lists of current and future trends, perceived by different groups of respondents to have an impact on an industry in the future [19,20]. With the method, we thus wanted to elicit which environmental trends were “top of mind” and rated as most important for different groups. We could then, in a second stage, compare and contrast responses between groups to look for any similarities. Contrary to how the Delphi method is commonly used, we did not seek to create a consensus between groups. Our results instead provide a crude illustration of the degree of overlap of mental maps between groups. Differences in trend perceptions between groups would indicate disagreement over which trends are important to react to, and could help explain why a firm finds it difficult to achieve consensus on the direction of more radical business model innovation. In the next section, we explain how we used the method in a step-by-step approach.

## Method steps

**Fig. 1:**
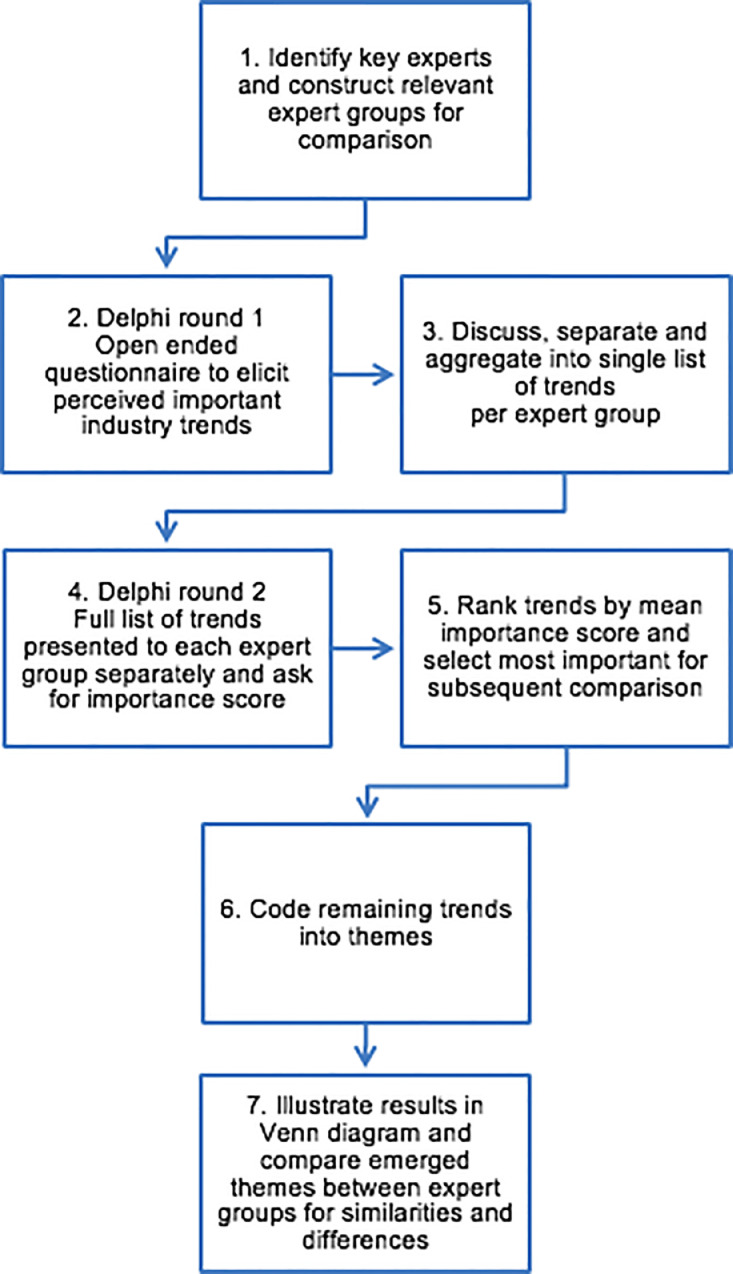
Stepwise methodology to extract and compare perceptions.

When designing the method, it seemed appropriate to examine shared rather than individual perceptions, since innovation decisions are typically also made at group level. An advantage of the Delphi method is that it allows for a broad investigation of a field of study, as it gathers knowledge from a variety of experts individually, which is then reflected upon by the whole panel. The proposed method was organized as a two-round Delphi-inspired survey as part of a process consisting of seven steps, as illustrated in Fig. 1. When developing the process, we specifically took inspiration from recommended Delphi procedures outlined by Schmidt [21] and by Okoli and Pawlowski [22], where the technique serves a dual purpose of soliciting opinions from experts and having them rank these according to importance.

In *step one*, key experts were identified, to construct relevant groups of experts to be compared. In our study we wished to compare three groups: (1) members of an innovation department, (2) members of the core business, and (3) an external expert group for reference. As the Delphi method is used to elicit knowledge from experts, the selection of appropriate experts is an important aspect of the method. The recommended size of each expert panel is 10–15 respondents, although less can be sufficient where the population size is small. Experts have to participate in several rounds, and it is important to inform them about the study form and, as far as possible, ask them to participate throughout the process. There will always be a risk of participant dropout in this kind of study, with typical dropout rates of 15–25% after each round reported in the literature. Therefore, when the experts are asked for commitment, it is important to try to be precise in the wording about what is expected of them and to give them an honest appraisal of their time commitment.

The researcher relies on receiving answers to the first round before the next round can be sent out. Therefore, it is also important to plan the interview phase in relation to invitation, deadlines for answers, and possible reminders.

In *step two*, a questionnaire was designed for use in a first Delphi round. This was administered separately for each group of experts, for as many groups as were included in the study (in our case three groups of experts). The initial questionnaire was very simple, consisting of an open-ended question, with the purpose to elicit knowledge about current and future trends perceived to have a significant impact on the firm. The question was formulated as follows: “*What current and future trends (*e.g. *social, political, economic, customer behavior, environmental, technological issues etc.) do you expect to have a significant impact on the industry in the future? Name and briefly explain as many trends as you find important*”. In this round, the experts were treated as individuals and the goal was to identify as many trends as possible (from every single expert).

In *step three*, the results of the first questionnaire were analyzed. The researchers discussed each trend separately. If some formulations were unclear, they were re-worded, with the aim of keeping respondents' statements as close to their own wording as possible. In cases where several trends were mentioned in one sentence, these were separated. When several respondents mentioned the same trends, these were merged. Finally, all identified trends were aggregated in one list. The outcome of step three is thus one list of trends for each group being studied (in our case three lists). To ensure reliability, where any rewording is necessary, at least two independent researchers should assess and discuss the responses. In the current case, we were two researchers to discuss each rewording. In addition, we asked an external colleague to independently verify that changes in wording made sense.

In *step four*, each group of experts were, in a random order, presented with the list of trends and asked to asses each trend on a Likert scale from 1 (not important) to 5 (very important). The objective of this second Delphi round was to present trends identified by the expert group, but allowing each respondent to assess the importance of each trend individually.

In *step five*, we ranked the trends by importance score. The answers will not necessarily be normally distributed, as there could be disagreement about the interpretation of the importance of a given trend. To find the trends interpreted as most important by each group, the list was reduced by selecting trends with a mean importance score greater than four. As the objective was to identify trends for which there was consensus about existence and importance within the expert group, choosing a higher cut-off point will naturally limit the list to the trends perceived to be most important for each group.

In *step six*, we compared the resulting lists for the groups to look for overlaps. This step was done as the process was run separately for each of the groups and a particular trend might be identified by several groups, but with a different wording. By performing a thematic analysis, we were able to create a final grouping of the trends found for all groups studied. Similarly, to step three, it is recommended that at least two independent researchers should asses and discuss the thematic analysis.

In *step seven*, to compare the degree of overlap in perceptions between the groups. In this last step, a Venn diagram was constructed in which the themes of trends are illustrated. This constitutes a qualitative visual illustration of the overlap of trends.

## Method validation with case

To illustrate our method we selected a case firm by screening the largest Danish firms, looking for those that (a) have a substantial market share in a core market, (b) have publicly announced having an innovation department, and (c) have publicly announced that they wish to engage with business model innovation. Quickly we identified a potential case, a market-leading Nordic non-life insurance firm. Initial informal interviews revealed that this firm was indeed pursuing business model innovation and faced some challenges regarding more radical forms of innovation. We therefore chose this firm as a suitable context for our study. The insurance industry is, like many other industries, highly dependent on the ability to adapt to rapidly changing trends. The insurance industry is known to be a very traditional business with a relatively conservative business model. The industry is nevertheless challenged in many ways, especially by new technologies. This includes self-driving cars, the spread of smart homes (the Internet of Things), and potential future competition from large IT firms like Google, who have access to a range of data that allows them to tailor insurance products.

The case firm has almost 3,400 employees and more than 3 million customers, with activities across Scandinavia. It offers a wide range of insurance products for the private, commercial and corporate markets, and each year handles almost 1 million claims. In 2016 they established a new dedicated innovation department, whose main purpose is to explicitly focus on both incremental and more radical forms of innovation, i.e. both incremental and radical, both product and business model innovation. In order to come up with new ideas, a big part of the work for the employees in the department is to monitor and identify new trends and technologies that can influence the industry.

**Fig. 2:**
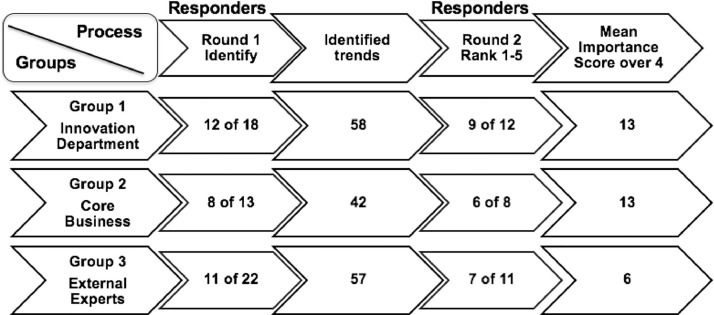
Group sizes, response rates, and trends found.

We used our method to elicit trends perceived to be important to three separate expert groups. We followed the process in Fig. 1, and the group sizes, response rates, and number of trends found are illustrated in Fig. 2 and described below. The first group comprised members of the innovation department of the case firm. We invited all 18 members of the innovation department to take part in the study. Of these, 13 answered the original questionnaire (step two). Based on their responses, 58 trends were identified (step three). Next the trends were presented to the 13 members who completed round one (step four). Nine of these answered (dropout rate of 30%).

The second group comprised members of the core business of the case firm. These were managers representing the various existing business areas in the organization. We chose managers who had a good knowledge of the core business of the firm. Thirteen people were invited to take part in the study. Of these, eight answered the questionnaire (step two). Based on their responses, 42 trends were identified (step three). Next the trends were presented to the eight managers who completed round one (step four). Of these, six answered (dropout rate of 25%).

The third group was composed of managers from competitors, industry consultants, analysts, and industry associations. Whilst the objective was mainly to compare two internal groups in the case firm, we composed this group in order to have an external reference, with which to compare the two internal groups. We initially contacted 22 industry experts. Eleven experts answered the questionnaire (step two). Based on their responses, 57 trends were identified (step three). Next, the trends were presented to the eleven experts (step four), of whom seven answered round two (dropout rate of 36%).

In step five, we removed trends with a mean importance score of 4 or below. This is a somewhat arbitrary cut-off point, equivalent to “important” on our Likert scale. The higher the cut-off, the fewer trends will emerge as important. The lower the cut-off, the more trends will be included for final analysis. Our cut-off yielded a total list of 32 trends, found in Table 1.

**Table 1:** Trends perceived to be most important for three expert groups (step five).

Trend	List of identified trends perceived to be of high importance by Innovation Department experts (mean importance score > 4)	Mean	Std. Dev
I3	We will increasingly see automated individualization of pricing based on data and AI (artificial intelligence)	4.44	0.73
I13	Algorithms will to a greater extent make decisions in connection with claims handling	4.44	0.73
I29	There will be higher expectations regarding the safe handling of data	4.44	0.88
I50	There will be an increase in regulation of companies' use of data	4.44	0.88
I8	There will be an increased use of customer data to customize products for customers	4.33	0.87
I12	There will be an increased threat of cyberattacks, hacking, data abuse, identity theft, etc.	4.33	1.00
I22	More existing service touch points will be automated	4.33	0.71
I4	We will see more self-driving cars on the roads	4.22	0.83
I7	Customers will expect greater transparency and responsibility in relation to the use of data	4.22	0.83
I36	The degree of self-service will increase in line with digital opportunities in modern society in general	4.22	0.83
I1	Technological developments will enable a more personal risk assessment	4.11	0.78
I21	Climate change will change types of damage	4.11	0.93
I23	We will increasingly see driverless technology; in the future, cars / buses will drive themselves	4.11	1.45

Trend	List of identified trends perceived to be of high importance by Core Business experts (mean importance score > 4)	Mean	Std. Dev
C5	Insurance company earnings from traditional premiums will decrease	4.67	0.52
C37	There will be increased automation (digitization / robotization) of claims processing	4.67	0.52
C21	There will be increased competition from both the financial sector and other entrants in the insurance market	4.50	0.55
C23	Insurance companies will increasingly use AI (artificial intelligence) for the purpose of taking advantage of existing customer data	4.50	0.55
C34	There will be an erosion of the industry's primary business due to falling traditional customer risks	4.33	0.82
C36	There is a trend towards increasingly outdated IT systems in the (insurance) industry	4.33	0.82
C38	It will be easier for consumers to report injuries	4.33	0.52
C41	More strategic partnerships will arise between insurance companies and partners with their own distribution – agreements between companies with shared value chains	4.33	0.52
C2	Standardization of claims management processes will be increased	4.17	0.98
C13	The pace/speed of technological development is enormous	4.17	0.75
C19	We will see more self-driving cars on the roads	4.17	0.75
C20	We will increasingly see smart homes	4.17	0.75
C28	Globalization will continue, leading to more global companies entering the Danish/Scandinavian market	4.17	0.75

Trend	List of identified trends perceived to be of high importance by External experts (mean importance score > 4)	Mean	Std. Dev
E12	In the future, insurance companies will have much more data available, for example from social media and connected devices, IoT (Internet of Things)	4.71	0.49
E32	We will increasingly see sales channels move towards digital channels and there will be more focus on being able to advise on and recommend products online	4.57	0.53
E3	In line with technological developments, companies will increasingly have customers serve themselves online	4.29	0.76
E37	Over time, we will see an increasing need for regulation regarding digital identities and data	4.29	0.49
E50	Customers are increasingly used to effective digital contact with companies from which they buy goods and services	4.29	0.76
E6	Self-driven cars are expected to cause fewer injuries (frequency of injury and personal injury)	4.14	0.69

In step six, a thematic analysis was performed by comparing the resulting lists for the groups, looking for overlaps. The resultant themes are found in Table 2.

**Table 2:** Themes resulting from grouping of trends (step 6).

Coded themes	Associated trends
*Themes linked to all three expert groups*	
1 Self-driving vehicles	I4, I23, C19, E6
2 More individualized assessment of risk and of product pricing thanks to technology	I1, I3, I8, C23, E12, E32
*Themes linked to two expert groups*	
3 Greater transparency and regulation in the use of data	I7, I29, I50, E37
4 Automation of service touch points and increased self-service	I22, I36, E3, E50
5 Increased automation in relation to decisions about insurance payouts in the future	I13, C37
*Themes linked to single expert group*	
6 Increased threat from cybercrime	I12
7 Change in types of damage due to climate change	I21
8 Increasing process standardization	C2
9 Enormous speed of technological development	C13
10 Growth in smart homes	C20
11 Lower margins on traditional premiums	C5, C34
12 Easier for customers to report claims	C38
13 Aging technology infrastructure in the industry	C36
14 Increased competition from e.g. financial sector	C21, C28
15 More strategic partnerships	C41

Several circumstances contribute to the validity of the method and the obtained data. It is important to carefully identify and select qualified experts within the area of the study. The anonymous nature of the study helps to ensure that the experts feel comfortable in reporting their thoughts and are not influenced by other experts, which can be the case in a face-to-face Delphi. In step four, trends were presented in a random order to avoid bias in the order of listing of the items. In relation to the interpretation of the results, in step three and six, to prevent that the results are influenced by preconceived ideas and prejudices, it is recommended that at least two independent researchers evaluate and discuss the results. Whilst these mentioned conditions increase the validity of the method, this increase is not quantifiable.

**Fig. 3:**
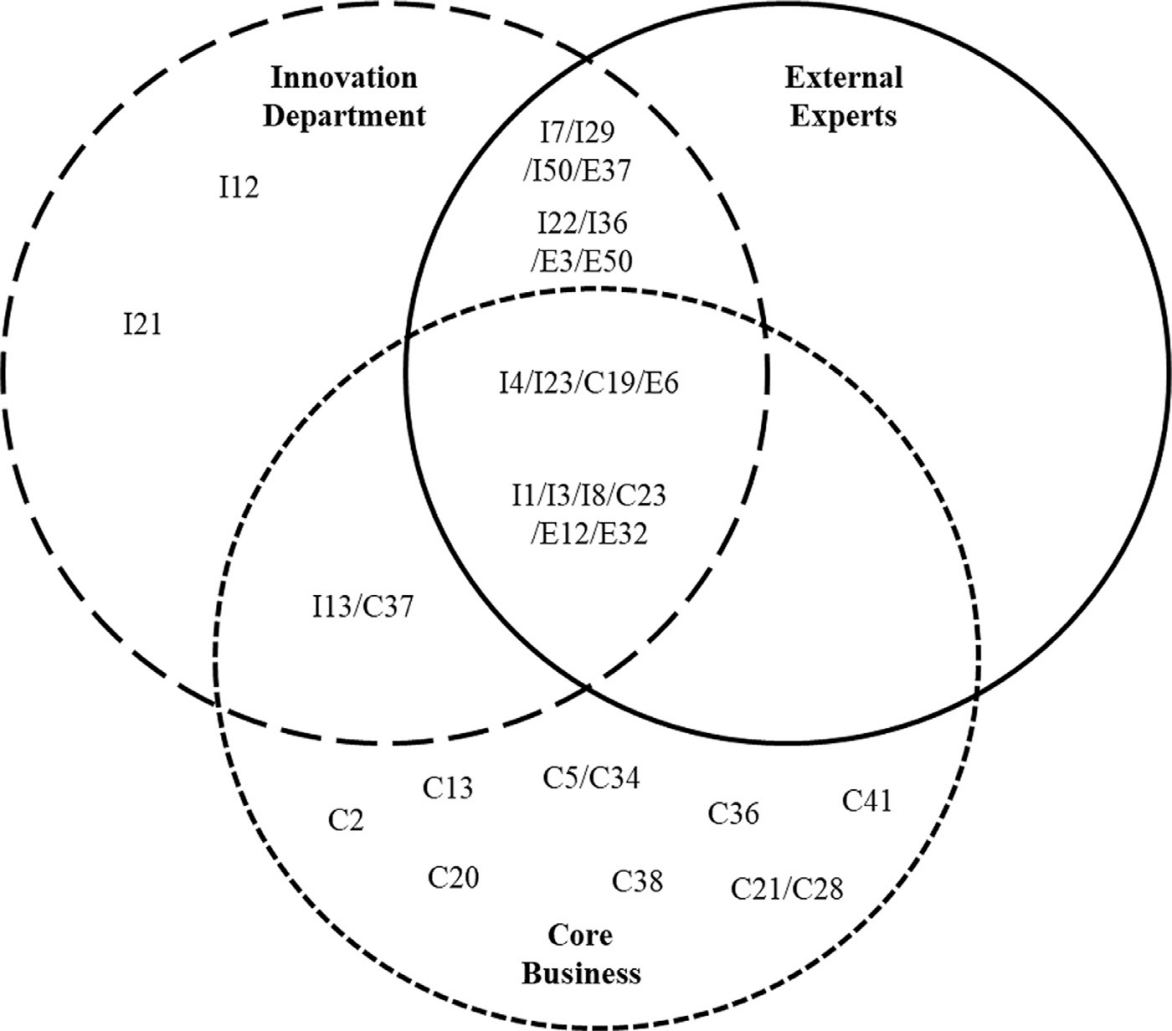
Venn diagram illustrating overlap of perceptions (step seven).

Finally, we created a Venn diagram in step seven, useful for visually representing the number of overlapping trends. The Venn diagram with associated themes is found in Fig. 3. In our particular study, the over-arching finding was that there was a high degree of similarity in trend perceptions between the innovation department and external experts. Core business managers, on the other hand, identified a range of trends not identified by the other two groups. We speculate that this is explained by the fact that the innovation department is tasked with keeping an eye on important trends in the external environment, thus noticing the same trends as the external experts we included in our study. The core business managers are tasked with maintaining daily operations, and thus do not have daily access to the same information environment as members of the innovation department.

## Lessons learnt

A number of practical issues emerged in the research process. One challenge was the relatively high dropout rates experienced after step four. We sent an invitation, followed by two reminders, all by email. This was done in the summer period. In a future study, we would recommend avoiding holiday periods. We would also recommend making telephone contact with the experts prior to sending out this round of questionnaires, in order to ensure commitment. Alternatively, one could make a phone call when an expert fails to respond to a first reminder. Another challenge related to this step was the size of the questionnaire. With 58, 42, and 57 trends to assess respectively, respondents would spend between 10 and 20 min to respond. The use of a Likert scale as opposed to, for example, a ranking, makes it cognitively easier for respondents to provide their judgment. Nevertheless, there is a risk of questionnaire fatigue.

## Declaration of Competing Interest

The authors have had full independence in carrying out this research and find no conflict of interest.
